# Techniques and Results for Open Hip Preservation

**DOI:** 10.3389/fsurg.2015.00064

**Published:** 2015-12-01

**Authors:** David M. Levy, Michael D. Hellman, Bryan Haughom, Michael D. Stover, Shane J. Nho

**Affiliations:** ^1^Department of Orthopedic Surgery, Hip Preservation Center, Rush University Medical Center, Chicago, IL, USA; ^2^Department of Orthopedic Surgery, Northwestern Memorial Hospital, Chicago, IL, USA

**Keywords:** hip preservation, open hip, periacetabular osteotomy, femoroacetabular impingement, dysplasia, Perthes, SCFE

## Abstract

While hip arthroscopy grows in popularity, there are still many circumstances under which open hip preservation is the most appropriately indicated. This article specifically reviews open hip preservation procedures for a variety of hip conditions. Femoral acetabular impingement may be corrected using an open surgical hip dislocation. Acetabular dysplasia may be corrected using a periacetabular osteotomy. Acetabular protrusio may require surgical hip dislocation with rim trimming and a possible valgus intertrochanteric osteotomy. Legg–Calve–Perthes disease produces complex deformities that may be better served with osteotomies of the proximal femur and/or acetabulum. Chronic slipped capital femoral epiphysis may also benefit from a surgical hip dislocation and/or proximal femoral osteotomy.

## Introduction

There are many hip pathomorphologies that cannot be addressed arthroscopically. Developmental or acquired deformities of the acetabulum (acetabular dysplasia, protrusio, or retroversion), femur (varus/valgus, torsion, or version), and femoral head or head–neck junction [Legg–Calve–Perthes disease (LCPD), chronic slipped capital femoral epiphysis, MED, MHE] may be more effectively treated with open hip preservation techniques. The present review article summarizes each of these pathomorphologic conditions and the clinical outcomes of techniques used to treat them.

## Femoroacetabular Impingement

Ganz and colleagues (1) were the first to describe the concept of femoroacetabular impingement (FAI). It is a pathologic process by which an intracapsular collision occurs between the femoral head–neck junction and the acetabular rim. Repetitive collision leads to labral injury, chondrolabral detachment, and degeneration (1–6). Two pathomorphologic categories are described: cam deformity of the proximal femur and pincer deformity of the acetabulum (1). Most often, these pathomorphologies coexist.

Labral and articular damage resulting from FAI is a source of pain and abnormal (dynamic) loading of the hip. Chondral flaps, labral tears, and loose bodies may produce locking and/or catching symptoms and labral tears theoretically disrupt the chondrolabral suction seal that provides constant fluid film lubrication to the joint (7). Patients complain of hip pain with flexion-based activities, such as sitting, squatting, stair climbing, and athletics. Most often pain is localized to the groin but may also be deep within the lateral, anterior, and posterior aspects of the hip, referred to as the “C-sign.” Impingement symptoms are most commonly reproduced with the “FADIR” maneuver: hip flexion to 90°, adduction, and internal rotation (8). Radiographic evaluation begins with various measurements on two-dimensional X-rays. The lateral center-edge angle of Wiberg and Tönnis angle are used to characterize acetabular morphology, while the α-angle and head–neck offset are used to characterize the femoral head–neck junction. A crossover sign showing the posterior acetabular rim crossing medial to the anterior rim, a prominent ischial spine sign showing intrusion of the ischial spine into the true pelvis, and a posterior wall sign showing the posterior rim passing medial to the center of the femoral head all may indicate global or focal acetabular retroversion (9).

**Figure 1:**
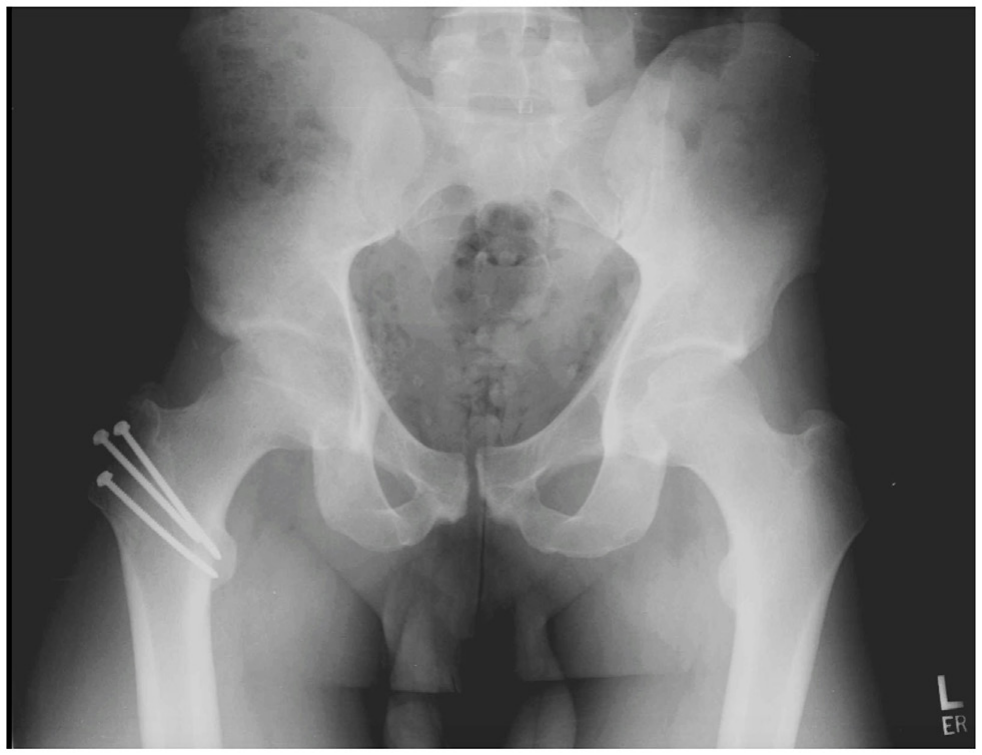
**A postoperative radiograph of a surgical hip dislocation secured with screws**.

Surgical hip dislocation has traditionally been the gold standard for treating FAI. Ganz et al. (10) was first to describe the currently accepted surgical technique. With the patient in a lateral decubitus position, the surgeon initiates the approach using either a Kocher–Langenbeck (KL) type or straight lateral incision. The fascial interval is developed by splitting the gluteus maximus (KL) or the Gibson interval, which spares the anterior half of the gluteus maximus (11). The anterior capsule is then accessed by a trigastric trochanteric osteotomy. The osteotomy can be performed with a step cut, which provides for greater stability and earlier progression of weight bearing (12). As the greater trochanter is osteotomized, the obturator externus muscle remains attached to the intact femur, protecting the deep branch of the MFCA, which is the primary blood supply to the femoral head (10, 12). An anterior Z-shaped capsulotomy followed by a transection of the round ligament facilitates an atraumatic anterior hip dislocation. Laser Doppler flowmetry has confirmed that perfusion to the femoral head is maintained after a trochanteric osteotomy and dislocation (12). The surgeon is left with a 360° view of both the acetabulum and the femur to perform osteochondroplasty and labral repair, debridement, or reconstruction. At the end of the procedure, the trochanter is reapproximated and stabilized with screws (see Figure 1). After surgery, patients must follow toe-touch weight-bearing restrictions for 4–8 weeks to allow for osteotomy healing.

Extensive literature has shown good to excellent outcomes following surgical hip dislocation for FAI. At an average of 4.7 years, Beck et al. (2) reported good to excellent Merle d’Aubigne hip scores in 13 of 19 patients who underwent surgical hip dislocation for FAI. Similarly, Kempthorne et al. (13) showed significant improvements in 53 patients’ Western Ontario and McMaster University Osteoarthritis Index (WOMAC) scores at 4-year follow-up. These promising outcomes also extend to the athletic population. In a case series of five professional ice hockey players, Bizzini et al. (14) successfully returned all players to full competition at an average of 9.6 months. Naal et al. (15) conducted a larger series of 22 mixed professional athletes and reported a 96% rate of return to competition. At a mean follow-up of 3.8 years, 82% of subjects were still satisfied with their hip surgery.

While the underlying FAI pathology is often successfully treated with open surgical dislocation, complications following this procedure are common with an overall complication rate reported around 6% (16). The most common complication is trochanteric bursitis secondary to prominent hardware at the osteotomy site (26%), followed by greater trochanter non-union (3–20%) and heterotopic ossification (3%) (17–20).

## Acetabular Dysplasia

Hip dysplasia usually starts during early childhood but may not manifest symptomatically until adolescence or young adulthood. An abnormally shallow, smaller acetabulum creates dysfunctional hip mechanics, labral shearing, and cartilage edge loading (21, 22). These pathologic alterations of the joint ultimately lead to early degeneration (23). Stulberg and Harris classically reported that 48% of patients with early degenerative hip arthritis had dysplastic features noted on radiographs (22). Patients most often complain of moderate-to-severe pain located in their groin, which occurs during daily activities. They will present with an antalgic limp, a positive impingement sign, and a positive Trendelenburg sign (24). Radiographically, the lateral center-edge angle of Wiberg is measured on an anteroposterior (AP) view, and the anterior center-edge angle is measured on a false profile view (9). An abduction-internal rotation AP view helps neutralize femoral anteversion, thus simulating the acetabular coverage that would be achieved by a proximal varus femoral osteotomy or a reorientation of the acetabulum (22).

**Figure 2:**
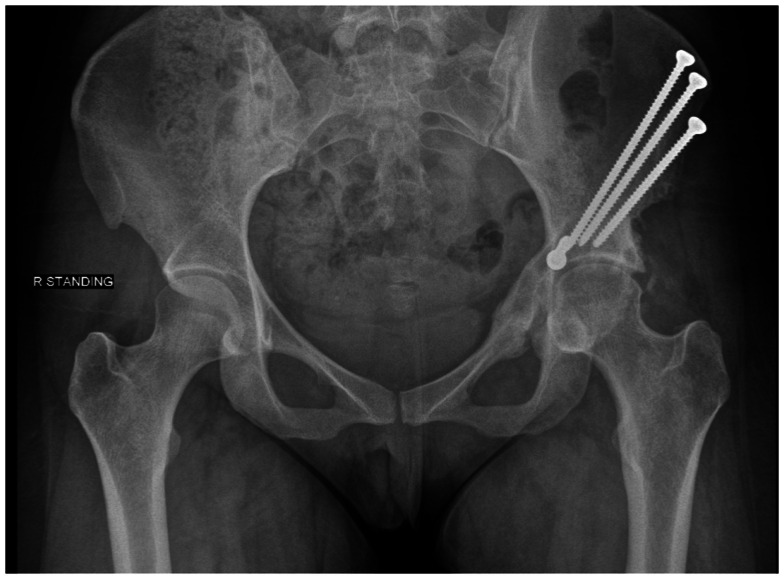
**A postoperative radiograph of a periacetabular osteotomy secured with screws**.

Numerous pelvic osteotomies have been designed to treat hip dysplasia. The primary goal of an osteotomy for dysplasia is to correct the deficiency in acetabular coverage. Reshaping osteotomies include the Pemberton and Dega techniques, which utilize incomplete cuts of the ilium to hinge off the triradiate cartilage. These osteotomies are reserved for skeletally immature individuals (22). Reconstructive osteotomies may be performed in patients with closed physes and utilize complete cuts of the pelvis in order to redirect joint loading forces. LeCouer first described the triple osteotomy in 1965 (22). This osteotomy requires two incisions to make individual cuts through the pubis, ischium, and ilium. The more commonly used technique today in adolescent and adult patients is the Bernese periacetabular osteotomy (PAO) (25). First described by Ganz, this procedure may be done through a single Smith-Peterson approach. It reorients the joint through four to five cuts done closer to the acetabulum than in the traditional triple osteotomy. These cuts maintain stability within the pelvis by not disrupting the posterior column. The osteotomy site is typically secured with screws (see Figure 2). Patients remain partial weight bearing with crutches for 6 weeks postoperatively (26).

Ganz et al. reported improvement in clinical outcomes and just one non-union in their original case series of 75 PAOs (25). Steppacher and colleagues rereviewed these 75 PAOs at 20-year follow-up. They found a 60.5% survivorship at 20 years with age and preoperative osteoarthritis grade being risk factors for failure (27). Matheney et al. (28) reviewed 135 PAOs after an average of 9-year follow-up. They reported 76% survival (defined as pain score under 10 and no conversion to arthroplasty). A recent systematic review revealed that a majority of the studies on PAO show significant improvements in radiographic parameters as well as clinical outcomes. The mean increase in anterior and lateral center-edge angles has ranged from 16 to 51° and 20 to 45°, respectively, and mean improvements in Harris Hip Scores have ranged from 14 to 33 points (29).

Major complications are common after PAO and occur in 3–37% of cases (28–30). The most common complications include heterotopic ossification, wound hematomas, transient neuropraxias (femoral and sciatic), inadvertent intra-articular extension, loss of fixation, and malreduction (17, 30). Non-union rates have been as high as 24% (31), and another 12–28% of patients require a reoperation due to symptomatic hardware (29). Surgeons must also be careful to avoid overcorrection, which could cause iatrogenic impingement.

Acetabular retroversion is an uncommon form of dysplasia that results in relative undercoverage of the posterior superior aspect of the femoral head. When the hip is flexed and adducted, the femoral head abuts the anterior wall, which causes pain and possibly induces posterior instability. The diagnosis is confirmed using the parameters described above (crossover sign, prominent ischial spine sign, and posterior wall sign), assessed on an AP pelvis radiograph. In cases of delayed presentation, the radiographs may also reveal fragmentation of the prominent anterior acetabular rim or an os acetabuli (21, 29).

The primary surgical option to correct acetabular dysplasia with retroversion is a reverse PAO. The osteotomy is performed the same as a Bernese PAO, utilizing a Smith-Peterson approach and 4–5 bone cuts (32). The free acetabular fragment is flexed and internally rotated before screws are used to secure it in place.

Siebenrock et al. (32) showed good to excellent results in 26 of 29 reverse PAOs with an average follow-up of 30 months. The average Merle d’Aubigne score improved from 14.0 to 16.9 and the crossover sign was eliminated in all except four patients. They revisited their cohort at 10 years and found that no patients were converted to total hip arthroplasty (THA) and there was no significant change in Tonnis osteoarthritis grades. Predictors for a poor outcome included not treating concomitant cam deformity and overcorrection of the acetabular version (33). Similarly, Peters et al. (26) showed a statistically significant improvement in Harris Hip Scores from 54 to 86 at 1-year follow-up and confirmed correction of radiographic retroversion in 96% of patients (31). These authors later developed an algorithmic approach in which they emphasized the importance of evaluating for a cartilaginous injury. Retroverted acetabuli without concomitant cartilaginous damage can be safely treated with a PAO; however, if magnetic resonance arthrography (MRA) confirms an injury to the articular cartilage, the authors recommend a surgical hip dislocation and acetabular rim debridement to avoid rotating diseased cartilage to a weight-bearing portion of the joint (34).

## Protrusio Acetabuli

Protrusio acetabuli is defined as a socket global overcoverage secondary to a relative medialization of the acetabulum. Protrusio is commonly found in patients with Marfan syndrome and inflammatory conditions like rheumatoid arthritis, but is most often considered to be idiopathic. If a cause is identified, attempted treatment of the underlying condition should precede any surgical intervention (35). A patient typically presents with groin pain and stiffness with daily activities. On an AP radiograph of the pelvis, the acetabular fossa and femoral head project medial to the ilioischial line (36). The decision to move forward with surgery depends on the patient’s age and amount of hip degeneration. Surgical options for patients without advanced protrusio include triradiate cartilage closure in skeletally immature patients and a valgus intertrochanteric osteotomy (VITO) with or without a global rim trimming in skeletally mature patients (35, 37, 38).

Steel (39) followed 19 skeletally immature patients who had undergone triradiate cartilage fusion for protrusio acetabuli. An anterior approach was used, followed by elevation of the obturator internus and exposure of the quadrilateral surface and triradiate cartilage. After triradiate closure, 12 of the 19 hips were radiographically graded as normal, four were downgraded to an “acetabular deepening,” and three showed no improvement. The only postoperative complication was transient femoral nerve palsy in one patient.

Surgical hip dislocation and/or a VITO are indicated for skeletally mature patients with protrusio acetabuli. A surgical hip dislocation facilitates labral repair or reconstruction and acetabular rim resection to reduce its depth (37). Some cases also require a femoral osteoplasty and relative femoral neck lengthening via a trochanteric advancement (40). A VITO procedure lateralizes the femur to restore normal mechanical alignment of the hip and facilitates femoroacetabular clearance. This procedure is especially indicated for patients with concomitant coxa vara, defined as a femoral neck-shaft angle <110°. With the patient-positioned supine, a direct lateral approach to the femur is used by splitting the vastus lateralis. A closing wedge of bone is removed and the lateral cortex of the proximal fragment is impacted into the distal fragment. The fragments are usually held together in this position using a blade plate. Toe-touch weight-bearing restrictions are enforced for the first 6 weeks after surgery (35, 41).

Results of a VITO procedure have been mixed. Rosemeyer et al. (41) reported good to excellent results in 21 of 25 hips at 6-year follow-up, while Hooper and Jones (38) reported fair to poor results in seven of nine patients at 2- to 7-year follow-up. In a more recent study, McBride et al. (35) presented 12 patients who underwent a VITO for 19 hips. While 83% of patients were satisfied with their decision to have surgery, eight of 19 hips (42%) were revised to THA between 10 months and 15 years after the index VITO. The authors stressed that osteotomy may not necessarily be the definitive treatment for protrusio patients but rather that it may delay the need for THA. They recommended that the procedure not be performed in individuals older than 40 years or who have significant arthritis. In this patient population, THA outcomes have been favorable as long as the acetabular component is lateralized with bone graft so that it aligns with the anatomic center (36, 42–44).

## Perthes-Like Diseases

Legg–Calve–Perthes disease is an idiopathic osteonecrosis of the capital femoral epiphysis in children. It most often affects children between 4 and 8 years old, boys far more commonly than girls. The residual deformity of LCPD after skeletal maturity leads to abnormal hip mechanics and may produce pain. Over 50% of patients develop symptomatic degenerative joint disease by the sixth decade of life (45). Deformity typically presents as a high-riding greater trochanter, a short femoral neck, and a misshapened/enlarged femoral head with variable acetabular dysplasia, retroversion, and joint incongruity (46, 47).

Surgical intervention should be reserved for symptomatic patients. Typical surgical treatment includes open hip dislocation with osteoplasty of the affected femoral head and labral repair. The remainder of the correction is tailored to the specific deformity present. In the setting of femoral retroversion, a proximal femur valgus derotational osteotomy procedure may be of benefit; if acetabular dysplasia is present, a PAO may be of benefit if instability exists following femoral surgery; and, in the setting of acetabular malrotation, an acetabular rim trimming may be of benefit (46, 47). Extra-articular impingement and abductor weakness may result from a high-riding greater trochanter. This can be corrected by advancing the trochanter distally and laterally, which (relatively) lengthens the femoral neck and increases the lever arm of the hip abductors (48). In addition, in LCPD patients with a shortened femoral head, the lesser trochanter may impinge on the ischium and posterior acetabulum. As with the greater trochanter, the lesser trochanter can also be advanced distally to allow clearance over the pelvis (48). Clohisy et al. (49) reported on their experience with surgical hip dislocation, femoral osteochondroplasty and PAO for Perthes-like conditions with acetabular dysplasia. Out of 16 patients with a minimum of 24-month follow-up, only two reported a modified Harris Hip Score (mHHS) <70 (considered a failure) with a median score of 92. They concluded that this procedure was both safe and effective for these less common deformities.

For LCPD patients with an abnormally widened head preventing a concentric joint, a femoral head reduction osteotomy may be performed (40). This is done through the same posterior approach and trochanteric flip osteotomy as described for a surgical hip dislocation. However, in order to visualize the intertrochanteric region, Ganz et al. have recommended an extended retinacular flap (50). The flap is created by subperiosteally dissecting the external rotators, MFCA, and superior retinacular vessels and reflecting them posteriorly until the base of the lesser trochanter is visible. Once adequate exposure is attained, a femoral head reduction osteotomy is performed with the goal of matching the superior femoral head’s contour with that of the inferior head. A trapezoidal segment of bone is removed from the superior head–neck junction with osteotomes in the sagittal plane (40). If the acetabulum does not fit properly over the reconstructed femoral head, it is advised to perform a PAO as well. All patients must remain toe-touch weight-bearing for 6–8 weeks (40). In a case series of 14 femoral head reduction osteotomies, Leunig and Ganz found that concomitant acetabular correction was needed in 13 of 14 patients but that no patients developed osteonecrosis after a minimum 3-year follow-up (40). All osteotomies had healed within 8 weeks. Complications are similar to the aforementioned osteotomies, including pseudoarthrosis, painful hardware, superficial infection, and reoperation.

## Slipped Capital Femoral Epiphysis

Slipped capital femoral epiphysis (SCFE) affects adolescent males at a 2:1 ratio compared to females. It is typically unilateral and more common in overweight African-American children. Failure at the hypertrophic zone of the capital physis allows the femoral neck to displace anteriorly and superiorly relative to the femoral epiphysis. Once diagnosed, *in situ* pinning is recommended to stop further slip progression and osteonecrosis. Nevertheless, even after fixation, the hip may remodel into an abnormal femoral head–neck junction causing impingement (51). Some authors have theorized that subclinical SCFE may be a cause of idiopathic FAI, but recent evidence has shown that classic cam deformities have a significantly different physeal tilt angle than SCFE deformities (52).

Surgical treatment for chronic SCFE depends on the severity of the deformity. A small slip angle can be treated with an arthroscopic femoral osteoplasty, but larger slips may require open techniques (53). Surgical hip dislocation with osteoplasty, femoral neck osteotomies, or intertrochanteric osteotomies can be employed. The intertrochanteric osteotomy as originally described by Imhauser in 1957 alters the lateral head-shaft angle to prevent impingement (51). After using a standard lateral approach to the femur, a wedge of bone is removed from the intertrochanteric region to create flexion, abduction, and internal rotation of the distal fragment. A blade plate is used to fix the osteotomy in place. After surgery, the hip is immobilized in flexion, abduction, and internal rotation for 8–12 weeks. Progressive weight bearing is usually allowed immediately after surgery (54).

Spencer et al. (55) demonstrated efficacy using both surgical methods for treating chronic SCFE deformities. Eleven of 13 patients who underwent surgical dislocation and osteoplasty alone either improved or were unchanged after a mean of 12 months, while five of six patients who had a combined osteoplasty and intertrochanteric osteotomy improved. All trochanteric osteotomies healed. Similarly, Rebello et al. (56) showed significant WOMAC improvements in 29 chronic SCFE deformities that underwent femoral osteoplasty and intertrochanteric osteotomy. Positive results continue over the long term as well. Kartenbender et al. (54) followed a cohort of 35 patients (39 hips) for an average of 23.4 years after intertrochanteric osteotomy. Nine hips had no pain and 22 hips had only slight pain with exercise, as 77% of patients were rated as good to excellent clinically (54). Schai et al. (57) reported the radiographic findings of 51 patients at an average of 24 years after osteotomy, showing that 55% had no degeneration, 28% had moderate degeneration, and 17% had severe osteoarthritis. Complication rates after the Imhauser intertrochanteric osteotomy have been as high as 48%. Acute joint space narrowing with a loss of motion may be seen in 29% of patients with a 5% loss of reduction and 10% delayed union rate (58).

For severe deformities, a subcapital realignment can be performed. After surgical hip dislocation, a second trochanteric osteotomy is made to develop a retinacular flap. The short external rotators along with the superior retinacular vessels are subperiosteally lifted until the entire femoral neck is exposed. Then an osteotomy is made through the remodeled physeal scar, the appropriate correction is made, and pins are placed in a retrograde fashion (59). Ziebarth and colleagues (60) showed that the operation was relatively safe and reported that 100% of their patients (40) had no evidence of osteonecrosis at 1-year follow-up. Anderson et al. (59) reported that at 61 months, the average change in Harris Hip Score was 23 (54–77). Complications occurred in four of the 12 cases with avascular necrosis in two patients (59).

## Conclusion

Open hip preservation techniques have been shown to relieve pain, improve function, and slow the progression of arthritis in adolescent and young adult patients with hip pathology. Larger, more complex deformities may require a more radical correction than can be achieved arthroscopically. All open techniques carry a significant risk of complications, but the surgeon must balance these risks with the benefits of surgery and individual circumstances of their patients. Overall, hip preservation can provide good to excellent outcomes, especially at major referral centers with a high volume of experience.

## Conflict of Interest Statement

The authors declare that the research was conducted in the absence of any commercial or financial relationships that could be construed as a potential conflict of interest.

## References

[1] Ganz R Parvizi J Beck M Leunig M Notzli H Siebenrock KA. Femoroacetabular impingement: a cause for osteoarthritis of the hip. Clin Orthop Relat Res (2003) 417:112–20. 10.1097/01.blo.0000096804.78689.c2 14646708

[2] Beck M Leunig M Parvizi J Boutier V Wyss D Ganz R. Anterior femoroacetabular impingement: part II. Midterm results of surgical treatment. Clin Orthop Relat Res (2004) 418:67–73. 10.1097/00003086-200401000-00012 15043095

[3] Larson CM Giveans MR Taylor M. Does arthroscopic FAI correction improve function with radiographic arthritis? Clin Orthop Relat Res (2011) 469(6):1667–76. 10.1007/s11999-010-1741-6 21181460 PMC3094626

[4] Agricola R Heijboer MP Bierma-Zeinstra SM Verhaar JA Weinans H Waarsing JH. Cam impingement causes osteoarthritis of the hip: a nationwide prospective cohort study (CHECK). Ann Rheum Dis (2013) 72(6):918–23. 10.1136/annrheumdis-2012-201643 22730371

[5] Lavigne M Parvizi J Beck M Siebenrock KA Ganz R Leunig M. Anterior femoroacetabular impingement: part I. Techniques of joint preserving surgery. Clin Orthop Relat Res (2004) 418:61–6. 10.1097/00003086-200401000-00011 15043094

[6] Barros HJ Camanho GL Bernabe AC Rodrigues MB Leme LE. Femoral head-neck junction deformity is related to osteoarthritis of the hip. Clin Orthop Relat Res (2010) 468(7):1920–5. 10.1007/s11999-010-1328-2 20352385 PMC2882000

[7] Ferguson SJ Bryant JT Ganz R Ito K. An in vitro investigation of the acetabular labral seal in hip joint mechanics. J Biomech (2003) 36(2):171–8. 10.1016/S0021-9290(02)00365-2 12547354

[8] Byrd JW. Evaluation of the hip: history and physical examination. N Am J Sports Phys Ther (2007) 2(4):231–40. 21509142 PMC2953301

[9] Clohisy JC Carlisle JC Beaule PE Kim YJ Trousdale RT Sierra RJ A systematic approach to the plain radiographic evaluation of the young adult hip. J Bone Joint Surg Am (2008) 90(Suppl 4):47–66. 10.2106/JBJS.H.00756 18984718 PMC2682767

[10] Ganz R Gill TJ Gautier E Ganz K Krugel N Berlemann U. Surgical dislocation of the adult hip a technique with full access to the femoral head and acetabulum without the risk of avascular necrosis. J Bone Joint Surg Br (2001) 83(8):1119–24. 10.1302/0301-620X.83B8.11964 11764423

[11] Gibson A Posterior exposure of the hip joint. J Bone Joint Surg Br (1950) 32-B(2):183–6. 15422015 10.1302/0301-620X.32B2.183

[12] Notzli HP Siebenrock KA Hempfing A Ramseier LE Ganz R. Perfusion of the femoral head during surgical dislocation of the hip. Monitoring by laser Doppler flowmetry. J Bone Joint Surg Br (2002) 84(2):300–4. 10.1302/0301-620X.84B2.12146 11922376

[13] Kempthorne JT Armour PC Rietveld JA Hooper GJ. Surgical dislocation of the hip and the management of femoroacetabular impingement: results of the christchurch experience. ANZ J Surg (2011) 81(6):446–50. 10.1111/j.1445-2197.2010.05489.x 22295348

[14] Bizzini M Notzli HP Maffiuletti NA. Femoroacetabular impingement in professional ice hockey players: a case series of 5 athletes after open surgical decompression of the hip. Am J Sports Med (2007) 35(11):1955–9. 10.1177/0363546507304141 17609527

[15] Naal FD Miozzari HH Wyss TF Notzli HP. Surgical hip dislocation for the treatment of femoroacetabular impingement in high-level athletes. Am J Sports Med (2011) 39(3):544–50. 10.1177/0363546510387263 21173196

[16] Sink EL Beaulé PE Sucato D Kim YJ Millis MB Dayton M Multicenter study of complications following surgical dislocation of the hip. J Bone Joint Surg Am (2011) 93(12):1132–6. 10.2106/JBJS.J.00794 21571987

[17] Beaule PE Le Duff MJ Zaragoza E. Quality of life following femoral head-neck osteochondroplasty for femoroacetabular impingement. J Bone Joint Surg Am (2007) 89(4):773–9. 10.2106/JBJS.F.00681 17403799

[18] Yun HH Shon WY Yun JY. Treatment of femoroacetabular impingement with surgical dislocation. Clin Orthop Surg (2009) 1(3):146–54. 10.4055/cios.2009.1.3.146 19885050 PMC2766742

[19] Zingg PO Ulbrich EJ Buehler TC Kalberer F Poutawera VR Dora C. Surgical hip dislocation versus hip arthroscopy for femoroacetabular impingement: clinical and morphological short-term results. Arch Orthop Trauma Surg (2013) 133(1):69–79. 10.1007/s00402-012-1616-2 23064993

[20] Papalia R Del Buono A Franceschi F Marinozzi A Maffulli N Denaro V. Femoroacetabular impingement syndrome management: arthroscopy or open surgery? Int Orthop (2012) 36(5):903–14. 10.1007/s00264-011-1443-z 22190060 PMC3337119

[21] Klaue K Durnin CW Ganz R The acetabular rim syndrome. A clinical presentation of dysplasia of the hip. J Bone Joint Surg Br (1991) 73(3):423–9. 1670443 10.1302/0301-620X.73B3.1670443

[22] Gillingham BL Sanchez AA Wenger DR. Pelvic osteotomies for the treatment of hip dysplasia in children and young adults. J Am Acad Orthop Surg (1999) 7(5):325–37. 10504359 10.5435/00124635-199909000-00005

[23] Murphy SB Ganz R Muller ME The prognosis in untreated dysplasia of the hip. A study of radiographic factors that predict the outcome. J Bone Joint Surg Am (1995) 77(7):985–9. 7608241 10.2106/00004623-199507000-00002

[24] Nunley RM Prather H Hunt D Schoenecker PL Clohisy JC. Clinical presentation of symptomatic acetabular dysplasia in skeletally mature patients. J Bone Joint Surg Am (2011) 93(Suppl 2):17–21. 10.2106/JBJS.J.01735 21543683

[25] Ganz R Klaue K Vinh TS Mast JW. A new periacetabular osteotomy for the treatment of hip dysplasias. Technique and preliminary results. Clin Orthop Relat Res (1988) 232:26–36. 3383491

[26] Peters CL Erickson JA Hines JL. Early results of the bernese periacetabular osteotomy: the learning curve at an academic medical center. J Bone Joint Surg Am (2006) 88(9):1920–6. 10.2106/JBJS.E.00515 16951106

[27] Steppacher SD Tannast M Ganz R Siebenrock KA. Mean 20-year followup of bernese periacetabular osteotomy. Clin Orthop Relat Res (2008) 466(7):1633–44. 10.1007/s11999-008-0242-3 18449617 PMC2505253

[28] Matheney T Kim YJ Zurakowski D Matero C Millis M. Intermediate to long-term results following the bernese periacetabular osteotomy and predictors of clinical outcome. J Bone Joint Surg Am (2009) 91(9):2113–23. 10.2106/JBJS.G.00143 19723987

[29] Clohisy JC Schutz AL St John L Schoenecker PL Wright RW. Periacetabular osteotomy: a systematic literature review. Clin Orthop Relat Res (2009) 467(8):2041–52. 10.1007/s11999-009-0842-6 19381741 PMC2706361

[30] Zaltz I Baca G Kim YJ Schoenecker P Trousdale R Sierra R Complications associated with the periacetabular osteotomy: a prospective multicenter study. J Bone Joint Surg Am (2014) 96(23):1967–74. 10.2106/JBJS.N.00113 25471911

[31] Peters CL Schabel K Anderson L Erickson J. Open treatment of femoroacetabular impingement is associated with clinical improvement and low complication rate at short-term followup. Clin Orthop Relat Res (2010) 468(2):504–10. 10.1007/s11999-009-1152-8 19885709 PMC2806994

[32] Siebenrock KA Schoeniger R Ganz R. Anterior femoro-acetabular impingement due to acetabular retroversion. Treatment with periacetabular osteotomy. J Bone Joint Surg Am (2003) 85-A(2):278–86. 12571306 10.2106/00004623-200302000-00015

[33] Siebenrock KA Schaller C Tannast M Keel M Buchler L. Anteverting periacetabular osteotomy for symptomatic acetabular retroversion: results at ten years. J Bone Joint Surg Am (2014) 96(21):1785–92. 10.2106/JBJS.M.00842 25378505

[34] Peters CL Anderson LA Erickson JA Anderson AE Weiss JA. An algorithmic approach to surgical decision making in acetabular retroversion. Orthopedics (2011) 34(1):10. 10.3928/01477447-20101123-07 21210626 PMC3399593

[35] McBride MT Muldoon MP Santore RF Trousdale RT Wenger DR. Protrusio acetabuli: diagnosis and treatment. J Am Acad Orthop Surg (2001) 9(2):79–88. 11281632 10.5435/00124635-200103000-00002

[36] Gates HS III Poletti SC Callaghan JJ McCollum DE. Radiographic measurements in protrusio acetabuli. J Arthroplasty (1989) 4(4):347–51. 10.1016/S0883-5403(89)80036-1 2621467

[37] Anderson LA Kapron AL Aoki SK Peters CL. Coxa profunda: is the deep acetabulum overcovered? Clin Orthop Relat Res (2012) 470(12):3375–82. 10.1007/s11999-012-2509-y 22898988 PMC3492622

[38] Hooper JC Jones EW Primary protrusion of the acetabulum. J Bone Joint Surg Br (1971) 53(1):23–9. 5578762

[39] Steel HH. Protrusio acetabuli: its occurrence in the completely expressed Marfan syndrome and its musculoskeletal component and a procedure to arrest the course of protrusion in the growing pelvis. J Pediatr Orthop (1996) 16(6):704–18. 10.1097/01241398-199611000-00002 8906639

[40] Leunig M Ganz R. Relative neck lengthening and intracapital osteotomy for severe Perthes and Perthes-like deformities. Bull NYU Hosp Jt Dis (2011) 69(Suppl 1):S62–7. 22035488

[41] Rosemeyer B Viernstein K Schumann HJ Follow up study of intertrochanteric valgus osteotomy with medial displacement in cases of primary protrusio acetabuly (author’s transl). Arch Orthop Unfallchir (1973) 77(2):138–48. 10.1007/BF00416446 4773145

[42] Ranawat CS Dorr LD Inglis AE. Total hip arthroplasty in protrusio acetabuli of rheumatoid arthritis. J Bone Joint Surg Am (1980) 62(7):1059–65. 7430191

[43] Bayley JC Christie MJ Ewald FC Kelley K. Long-term results of total hip arthroplasty in protrusio acetabuli. J Arthroplasty (1987) 2(4):275–9. 10.1016/S0883-5403(87)80059-1 3430153

[44] Heywood AW. Arthroplasty with a solid bone graft for protrusio acetabuli. J Bone Joint Surg Br (1980) 62(3):332–6. 7410465 10.1302/0301-620X.62B3.7410465

[45] McAndrew MP Weinstein SL A long-term follow-up of Legg-Calve-Perthes disease. J Bone Joint Surg Am (1984) 66(6):860–9. 6736087 10.2106/00004623-198466060-00006

[46] Sink E Zaltz I Session Participants Report of break-out session: management of sequelae of Legg-Calve-Perthes disease. Clin Orthop Relat Res (2012) 470(12):3462–3. 10.1007/s11999-012-2623-x 23054517 PMC3492617

[47] Albers CE Steppacher SD Ganz R Siebenrock KA Tannast M Joint-preserving surgery improves pain, range of motion, and abductor strength after Legg-Calve-Perthes disease. Clin Orthop Relat Res (2012) 470(9):2450–61. 10.1007/s11999-012-2345-0 22528379 PMC3830093

[48] Kelikian AS Tachdjian MO Askew MJ Jasty M Greater trochanteric advancement of the proximal femur: a clinical and biomechanical study. Hip (1983):77–105. 6671922

[49] Clohisy JC Nepple JJ Ross JR Pashos G Schoenecker PL. Does surgical hip dislocation and periacetabular osteotomy improve pain in patients with Perthes-like deformities and acetabular dysplasia? Clin Orthop Relat Res (2015) 473(4):1370–7. 10.1007/s11999-014-4115-7 25560960 PMC4353550

[50] Ganz R Huff TW Leunig M. Extended retinacular soft-tissue flap for intra-articular hip surgery: surgical technique, indications, and results of application. Instr Course Lect (2009) 58:241–55. 19385538

[51] Aronsson DD Loder RT Breur GJ Weinstein SL. Slipped capital femoral epiphysis: current concepts. J Am Acad Orthop Surg (2006) 14(12):666–79. 17077339 10.5435/00124635-200611000-00010

[52] Monazzam S Bomar JD Dwek JR Hosalkar HS Pennock AT. Development and prevalence of femoroacetabular impingement-associated morphology in a paediatric and adolescent population: a CT study of 225 patients. Bone Joint J (2013) 95-B(5):598–604. 10.1302/0301-620X.95B5.30118 23632667

[53] Azegami S Kosuge D Ramachandran M. Surgical treatment of femoroacetabular impingement in patients with slipped capital femoral epiphysis: a review of current surgical techniques. Bone Joint J (2013) 95-B(4):445–51. 10.1302/0301-620X.95B4.30245 23539694

[54] Kartenbender K Cordier W Katthagen BD Long-term follow-up study after corrective Imhauser osteotomy for severe slipped capital femoral epiphysis. J Pediatr Orthop (2000) 20(6):749–56. 10.1097/01241398-200011000-00010 11097248

[55] Spencer S Millis MB Kim YJ. Early results of treatment of hip impingement syndrome in slipped capital femoral epiphysis and pistol grip deformity of the femoral head-neck junction using the surgical dislocation technique. J Pediatr Orthop (2006) 26(3):281–5. 10.1097/01.bpo.0000217726.16417.74 16670535

[56] Rebello G Spencer S Millis MB Kim YJ. Surgical dislocation in the management of pediatric and adolescent hip deformity. Clin Orthop Relat Res (2009) 467(3):724–31. 10.1007/s11999-008-0591-y 19002743 PMC2635463

[57] Schai PA Exner GU Hansch O. Prevention of secondary coxarthrosis in slipped capital femoral epiphysis: a long-term follow-up study after corrective intertrochanteric osteotomy. J Pediatr Orthop B (1996) 5(3):135–43. 10.1097/01202412-199605030-00001 8866276

[58] Salvati EA Robinson JH Jr O’Down TJ. Southwick osteotomy for severe chronic slipped capital femoral epiphysis: results and complications. J Bone Joint Surg Am (1980) 62(4):561–70. 7380857

[59] Anderson LA Gililland JM Pelt CE Peters CL. Subcapital correction osteotomy for malunited slipped capital femoral epiphysis. J Pediatr Orthop (2013) 33(4):345–52. 10.1097/BPO.0b013e31827d7e06 23653020 PMC3648794

[60] Ziebarth K Zilkens C Spencer S Leunig M Ganz R Kim YJ. Capital realignment for moderate and severe SCFE using a modified Dunn procedure. Clin Orthop Relat Res (2009) 467(3):704–16. 10.1007/s11999-008-0687-4 19142692 PMC2635450

